# One of the many faces of COVID-19 infection: an irregularly shaped pulmonary nodule

**DOI:** 10.1186/s13244-021-00987-7

**Published:** 2021-04-16

**Authors:** Sevtap Arslan, Emre Ünal

**Affiliations:** Department of Radiology, Hacettepe University School of Medicine, 06100 Ankara, Turkey

Dear editor,

We read with great interest the educational review article titled ‘COVID-19 pneumonia: the great radiological mimicker’ by Duzgun et al. [1] in the November 2020 issue of Insights into Imaging. We agree with the authors that COVID-19 infection may mimic other airspace disorders on imaging. It is well known that the most common computed tomography (CT) finding of COVID-19 pneumonia is bilateral ground-glass opacity (GGO) with accompanying consolidation. However, the differentiation of COVID-19 infection with other disorders may not be straightforward due to various imaging findings. The incidence rate of irregular shaped solid nodules on CT scans of patients with COVID-19 infection has been reported as 3–12% in the literature [2–4]. We would like to share a challenging case of COVID-19 pneumonia presented with unusual imaging findings.

A 57-year-old smoker male patient presented to the emergency department with a 4-day history of cough and joint pain. His past medical history was unremarkable except for chronic kidney disease. Physical examination revealed abnormal lung sounds. The subsequent blood tests demonstrated lymphopenia (700/µl) and elevated level of C-reactive protein (10 mg/l). Chest CT was performed following polymerase chain reaction (PCR) test positivity for COVID-19 infection. An irregularly shaped solid nodule 2 cm in diameter was found in left upper lobe of the lung along with the CT findings compatible with COVID-19 pneumonia (Fig. 1a, b). Primary lung cancer could not be excluded with the imaging findings per se, in a smoker patient. According to the Fleischner Society 2017 guidelines [5], percutaneous transthoracic core needle biopsy was planned after the quarantine period ended. On pre-procedure CT scan, the nodule appeared to be decreasing in size with associated subpleural lines (Fig. 1c). Percutaneous biopsy procedure was avoided, and a follow-up CT was recommended. The follow-up CT scan obtained after 3 months revealed complete resolution of the nodule (Fig. 1d).

**Fig. 1:**
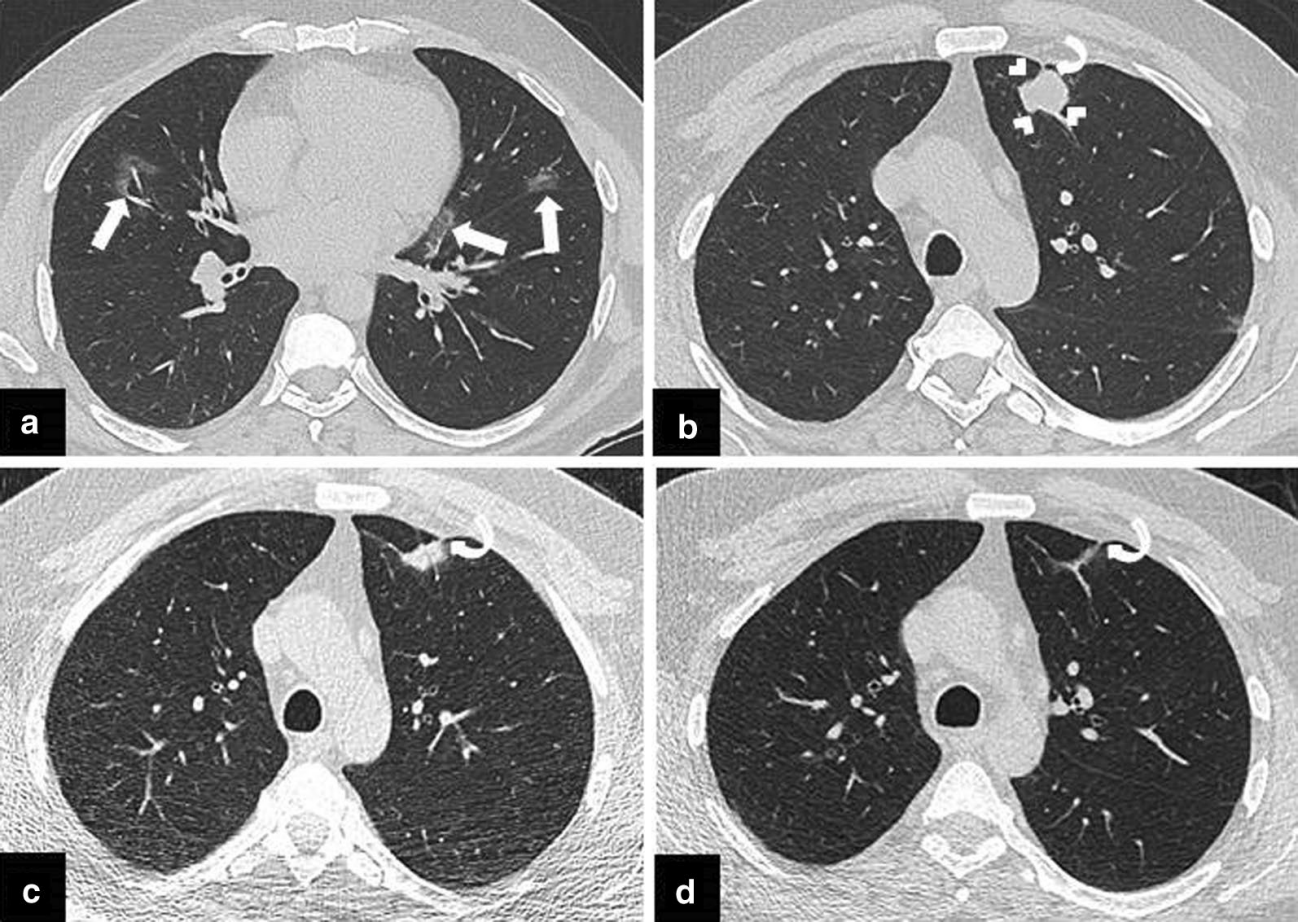
**a**, **b** The initial chest CT scan obtained following PCR test positivity for COVID-19 infection, revealed a few patchy areas of ground glass opacity (GGO) in both lungs (arrows) compatible with COVID-19 pneumonia. An irregularly shaped solid nodule 2 cm in diameter in left upper lobe of the lung was also noted (arrowheads). Percutaneous transthoracic core needle biopsy was scheduled due to suspicion of primary lung cancer. **c** CT scan obtained prior to biopsy procedure demonstrated significant size reduction of the nodule. Therefore, biopsy was not performed. **d** Follow-up CT scan obtained 3 months later demonstrated complete resolution of the nodule. A pleural tag which became more apparent following resolution of the nodule (curved arrows, **b**–**d**) raised the suspicion of COVID-19 triggered focal organizing pneumonia

In conclusion, CT is an indispensable tool in patients with clinical suspicion of COVID-19 infection. Imaging plays a significant role in the diagnosis and also evaluation of treatment response in COVID-19 infection. However, COVID-19 infection may result in various imaging findings since it is the great radiological mimicker as reported by Duzgun et al. [1]. Despite being rare, solitary pulmonary nodules with irregular margins are one of the many faces of COVID-19 infection. In the presented case, a pleural tag which gives rise to suspicion of organizing pneumonia was also observed on CT [6]. Spontaneously regressing solitary pulmonary nodule may be associated with organizing pneumonia which has been shown to occur secondary to COVID-19 infection [7].

## Data Availability

Data sharing is not applicable to this article as no datasets were generated or analyzed during the current study.

## Abbreviations

COVID-19: Coronavirus disease 2019
CT: Computed tomography
GGO: Ground-glass opacity
PCR: Polymerase chain reaction
